# Integrated Analysis of Omics Data Reveal AP-1 as a Potential Regulation Hub in the Inflammation-Induced Hyperalgesia Rat Model

**DOI:** 10.3389/fimmu.2021.672498

**Published:** 2021-05-28

**Authors:** Xiang Zhu, Feng Li, Miqun Wang, Huibin Su, Xuedong Wu, Haiyan Qiu, Wang Zhou, Chunli Shan, Cancan Wang, Lei Wei

**Affiliations:** ^1^Department of Anesthesiology, Affiliated Hospital of Nantong University, Nantong, China; ^2^The Yancheng Clinical College of XuZhou Medical University, The First People's Hospital of Yancheng, Yancheng, China; ^3^Department of Anesthesiology, Qingdao Women and Children’s Hospital, Qingdao, China; ^4^Department of Anesthesiology, Suzhou Municipal Hospital Affiliated to Nanjing Medical University, Suzhou, China; ^5^Department of Anesthesiology, The First People’s Hospital of Yancheng, Yancheng, China; ^6^Graduate School of Nantong University, Nantong, China

**Keywords:** chronic pain, transcriptomic (RNA-Seq), DNA methylation, multi-omics, AP-1

## Abstract

Inflammation-associated chronic pain is a global clinical problem, affecting millions of people worldwide. However, the underlying mechanisms that mediate inflammation-associated chronic pain remain unclear. A rat model of cutaneous inflammation induced by Complete Freund’s Adjuvant (CFA) has been widely used as an inflammation-induced pain hypersensitivity model. We present the transcriptomics profile of CFA-induced inflammation in the rat dorsal root ganglion (DRG) *via* an approach that targets gene expression, DNA methylation, and post-transcriptional regulation. We identified 418 differentially expressed mRNAs, 120 differentially expressed microRNAs (miRNAs), and 2,670 differentially methylated regions (DMRs), which were all highly associated with multiple inflammation-related pathways, including nuclear factor kappa B (NF-κB) and interferon (IFN) signaling pathways. An integrated analysis further demonstrated that the activator protein 1 (AP-1) network, which may act as a regulator of the inflammatory response, is regulated at both the transcriptomic and epigenetic levels. We believe our data will not only provide drug screening targets for the treatment of chronic pain and inflammation but will also shed light on the molecular network associated with inflammation-induced hyperalgesia.

## Introduction

Chronic pain, which affects more than 17% of all Canadians and approximately 100 million people in the United States (1, 2), represents one of the most common and distressing issues, with significant influences on society and individuals (3). Chronic pain is typically accompanied by inflammation, tissue damage, and hyperalgesia, which is a separate condition from chronic pain (4). Risk factors for chronic pain can include sociodemographic, mental, clinical, and biological factors (5). To date, inflammation and tissue damage are widely believed to represent the leading biological factors that induce chronic pain (6, 7). However, more studies examining the pathogenesis of chronic pain at the molecular level remain necessary.

Animal inflammation models have been widely used to assess the production of inflammatory mediators. A rat model of cutaneous inflammation induced by Complete Freund’s Adjuvant (CFA) has been widely characterized in the literature and has long been used to identify novel anti-inflammatory molecules (8). Swelling, hyperalgesia, and allodynia can be used to evaluate the inflammatory response in the CFA-induced model (8). The long-term hyperexcitability of primary sensory neurons in the dorsal root ganglion (DRG) and secondary sensory neurons in the spinal cord dorsal horn (SDH) is considered to be the underlying mechanisms for altered pain perception in the CFA model (9). CFA-induced chronic inflammation causes chronic pain, and the CFA-induced rat model of chronic pain is among the most commonly used animal models of chronic pain (10–13). However, the mechanism through which CFA induces chronic pain is not yet fully understood.

Recently, the integrated analysis of multi-omics data, which relies on next-generation sequencing (NGS), has revealed numerous complex molecular networks that underlie basic biological processes and human diseases (14, 15). DNA, RNA, and protein work synergistically to perform all biological functions. We have focused on chronic pain studies for years and are extremely interested in understanding the underlying biological networks through which CFA induces chronic pain in animals. A pioneering study was published by Tan et al. in 2017, in which they used an array-based analysis focused on the microRNA (miRNA) and mRNA network to reveal that several miRNA-mRNA interactions contributed to the inflammatory pain process. For example, they found that miR-124-3p could attenuate inflammatory pain and decrease interleukin-6 receptor (IL-6R) expression in the spinal cord (16). In addition to the findings from that study, miRNAs have been widely associated with various inflammatory models (17–20). The inhibition of some miRNAs has been considered to represent novel treatment strategy for chronic pain (21–23). However, focusing only on post-transcriptional regulation is not sufficient to illustrate the comprehensive regulation of CFA-induced inflammatory processes. Our previous work has demonstrated that the dysregulation of DNA methylation is involved in the CFA-induced inflammation model (11). A recent study also indicated that the hypomethylation of nerve growth factors could influence inflammatory hyperalgesia in rats (24). These studies have suggested that epigenetic regulations, especially DNA methylation, could contribute to inflammatory pain. In addition, although Tan et al. reported rat SDH tissue expression profiles for days after CFA treatment using microarray technology (16), the transcriptomic profiles of DRG tissues from chronic pain animal models remain lacking, indicating that the effects of CFA treatment on gene expression in the primary sensory neurons are still not clear.

To provide a comprehensive profile of the transcriptomic response to CFA in DRG tissues, we used an approach that targeted DNA methylation, gene transcription, and post-transcriptional regulation, including mRNA sequencing (mRNA-seq), small-RNA-seq, and targeted bisulfite sequencing, to detect alterations at the transcriptional level. We identified 418 differentially expressed genes (DEGs), 120 differentially expressed miRNAs and 2,670 differentially methylated regions (DMRs), which were associated with multiple inflammation-related pathways. An integrated analysis further indicated that activator protein 1 (AP-1), a key regulator of the inflammatory response, is regulated at the both the transcriptomic and epigenetic levels.

## Methods

### Animal Model

A total of 30 adult, male, specific pathogen-free (SPF)-grade Sprague Dawley (SD) rats (15 pairs) weighing approximately 200 ± 5 g were purchased from the Experimental Animal Center of Suzhou University. The SD rats were maintained in a standardized feeding environment under a 12-hour light-dark cycle at an ambient temperature of 24 ± 1°C. The CFA-induced chronic inflammatory pain model was established through unilateral subcutaneous injections of 0.1 mL CFA into the sterile foot skin, as described in our previous research (8, 11). The following experiments were performed in 10 pairs of rats (CFA and control), whereas the remaining 5 pairs of rats were used for the validation experiments. All experiments were approved by the Animal Research Ethics Committee of The First People’s Hospital of Yancheng, Suzhou Municipal Hospital Affiliated to Nanjing Medical University and Nantong University. Mechanical and thermal pain threshold measurements were also performed, as described in detail in our previous study (11).

### Mechanical Pain Threshold Measurement

The paw withdrawal threshold (PWT) was measured through using the von Frey filament needle (0.4 g‐15.0 g) to evaluate the mechanical attack of rats. The rats were first placed in a self‐made plexiglass box of which size is 22 cm×12 cm×22 cm, at duration of 1 hours per day for 3 days. The adaptation process mimics the normal living environment of rats. Pain threshold was measured while keeping surrounding environment quiet.

### Thermal Pain Thresholds Measurement

The paw withdrawal latency (PWL) was measured by heat radiation method for the thermal hyperalgesia of rats. The plexiglass box was placed on a glass plate of which thickness is 3 mm. First, the rats were placed in the glass box for 30 minutes, then their feet were irradiated with a heat stimulator according to the Hargreaves method. The time from the beginning of the irradiation to the emergence of leg lift to avoid the heat shrinkage was defined as PWL. In order to prevent tissue damages, the automatic cut‐off time was set to 20 seconds. Each rat was repeatedly tested for 5 times with intervals of more than 3 minutes. All behavioral experiments were performed under double‐blind conditions.

### RNA-Seq, Small-RNA-Seq, and Targeted Bisulfite Sequencing

For the NGS-based sequencing, DRG tissues from 10 randomly selected pairs of CFA-treated and untreated rats were extracted. The RNA-seq library was prepared with NEBNext Ultra RNA with Poly-A selection and was sequenced on an Illumina Hi-Seq 4000. The small RNA-seq library was constructed with NEBNext Multiplex Small RNA Library Prep Kit for Illumina (#7560S). The targeted bisulfite sequencing library was prepared with a DNA library construction TruSeq DNA LT Sample Prep Kit v2 for Illumina. The C-T transition was performed using a C-T transition EpiTect Bisulfite kit from Qiagen (Germany).

### RNA Sequencing Analysis

After running fastQC (0.11.8) for quality control, Cutadapt (2.1.0) was used to trim adapters and low-quality sequences (Phred score less than 20) from the raw fastq files. The cleaned fastq files were then mapped to the rn4 rat reference genome using STAR (2.7.0) with the default settings. Differential expression analysis was then performed by DEseq2 (1.29.0). Significant DEGs were defined by a p-value ≤ 0.05 and a log_2_ fold change ≥ 0.5. Further pathway enrichment analysis was performed with EnrichR. The gene set enrichment analysis (GSEA) was performed by using javaGSEA2-3.0.

The RNA-seq wig file generated by STAR was then used as the input for DaPars (0.9.1) to identify alternative polyadenylation events. Significant events were defined by a p-value < 0.05 and an absolute delta PDUI > 0.1 (25).

### Small RNA Sequencing Data Analysis

Small RNA-seq data were analyzed using the sRNAnalyzer pipeline (26). Adapter sequences were provided as inputs to the pipeline to remove adapter contamination. The cleaned reads were then mapped to a rat miRNA database to generate a count matrix, which was further analyzed by DEseq2 to identify differentially expressed miRNAs. Significantly expressed miRNA were defined based on a p-value < 0.05. The miRNA targets were predicted using ‘Multimir’ (1.10.0) R package. Finally, the pathway enrichment analysis was performed by using EnrichR.

### DNA Methylation Data Analysis

TrimGalore (0.6.4) was used for sequencing data quality control, adapter removal, and the trimming of low-quality sequences (Phred score < 20). The cleaned data was then mapped to the rn4 rat reference genome using Bismark (0.22.0). The generated CpG methylation ratios were then used as inputs for metilene to call DMRs (27). Significant DMRs were defined by p-values < 0.05 and absolute methylation ratio changes > 0.1. Significant DMRs were annotated to the nearest genes using Homer (4.8). Pathway enrichment analysis and motif enrichment analysis were performed by EnrichR and MEME-suite, respectively.

### Statistical Analysis

Enrichment analysis was detected by Fisher’s exact test, and p-values < 0.05 were considered significant. False discovery rate (FDR) correction was used to generate p-adj values for all expression matrix data (RNA-seq, small-RNA-seq, and DNA methylation). All data are expressed as the mean ± standard error (SE) and were analyzed with a Student’s t-test. A p-value < 0.05 was considered significant.

### Quantitative Real-Time PCR Assays

DRG tissues from 4 pairs of rats (control and CFA) were separated into halves, and one half was used for quantitative real-time PCR (qPCR) assays. Total RNA was extracted from cells using TRIpure reagents (Bioteke Beijing) and then reverse-transcribed using a first-strand cDNA synthesis kit (TSK302S, RT6 cDNA Synthesis Kit Ver 2). Reverse-transcribed products were used as templates for qPCR using the 2×T5 Fast qPCR Mix (SYBR Green I). The primers used for different target genes were as follows: Reg3a: F2:CTGTCAACCGTGGTAACTGTGG; Reg3a: R2: TGCGGGTCTACTGCTTGAACT; Reg3b: F:TCAACTGGGAGAGGAACCCA; Reg3b: R:TTGCCCCTTGACAGGATGTG; Dusp1: F2:AACGAGGCGATTGACTTTATAGACT; Dusp1: R2:TCCGCCTCTGCTTCACGA; Egr1: FAACCCTACGAGCACCTGACC; Egr1: R:AAGCGGCCAGTATAGGTGATG; rno-miR-384-3p: RT: GTCGTATCGACTGCAGGGTCCGAGGTATTCGCAGTCGATACGACATTGTG; rno-miR-384-3p: F:CCGGCATTCCTAGAAATTGTT; rno-miR-181b-5p: RT : GTCGTATCGACTGCAGGGTCCGAGGTATTCGCAGTCGATACGACACCCAC; rno-miR-181b-5p: F:GGCAACATTCATTGCTGTCG; common: R:ACTGCAGGGTCCGAGGTATT; rat actin: F:AGATCAAGATCATTGCTCCTCCT; rat actin: R:ACGCAGCTCAGTAACAGTCC.

### Western Blotting

DRG tissues from 3 pairs of mice were collected for protein extraction (total n = 6). DRG tissues were washed 3 times before the tissue preparation. Total lysates were separated by sodium dodecyl sulfate-polyacrylamide gel electrophoresis (SDS-PAGE) and then transferred for 1 hour to nitrocellulose (NC) membranes. The membranes were then blocked for 45 min with Tris-buffered saline containing Tween 20 (TBST) containing 3% (mass/vol) nonfat dry milk. Antibodies for Early Growth Response 1 (Egr1) were purchased from Invitrogen. Antibody for α-actin was purchased from Cell Signaling. After the incubation with primary antibodies (1:1000), the membranes were washed and then incubated with HRP-conjugated anti-mouse or anti-rabbit secondary antibodies (1:10000). The membrane was then visualized using enhanced chemiluminescence (ECL) western blotting substrate (Bio-Rad). Quantitative analysis of western blot results was performed using ImageJ software.

### Luciferase Assays

The AP-1 luciferase plasmid was purchased from BPS Bioscience (BPS Bioscience #79823). The injection of the luciferase reporter plasmid was performed according to the protocol published by Wang et al. in 2005 (28). The expression of the luciferase reporter gene was examined using a luciferase activity assay. Rats (n = 5) were sacrificed at 36 hours post-CFA treatment and luciferase injection. The tissues were collected, lysed, and assayed for luciferase activity using the luciferase system (Promega).

## Results

### CFA-Induced Inflammation Rat Model

**Figure 1:**
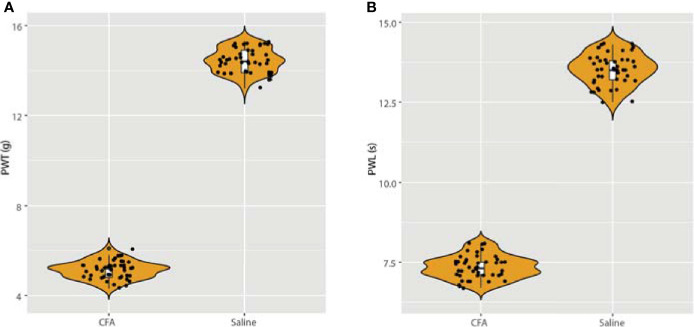
Detection of paw withdrawal threshold (PWT) and paw withdrawal latency (PWL) after injection of CFA. **(A)** After 24 h of CFA injection, the rats in the model group presented with mechanical hyperalgesia, n = 15. **(B)** After injection of CFA for 24 h, the PWL was also measured, which validated our previous model (11).

To establish the CFA-induced inflammation rat model, we injected male SD rats (200 ± 5 g body weight) with 0.1 mL CFA on the plantar skin of the right hind paw. The detailed methods are described in the Methods section and were published in our previous work. At 24 hours after the CFA injection, we measured the rat hind paw withdrawal threshold (PWT) and the paw withdrawal latency (PWL) in each group. As expected, we discovered a significant downregulation in both the PWT and PWL in the CFA group compared with the saline-treated control group (**Figure 1**). These results also validated our previous experiments (11).

### RNA-Seq Profiling of the CFA-Induced Inflammation Rat Model

**Figure 2:**
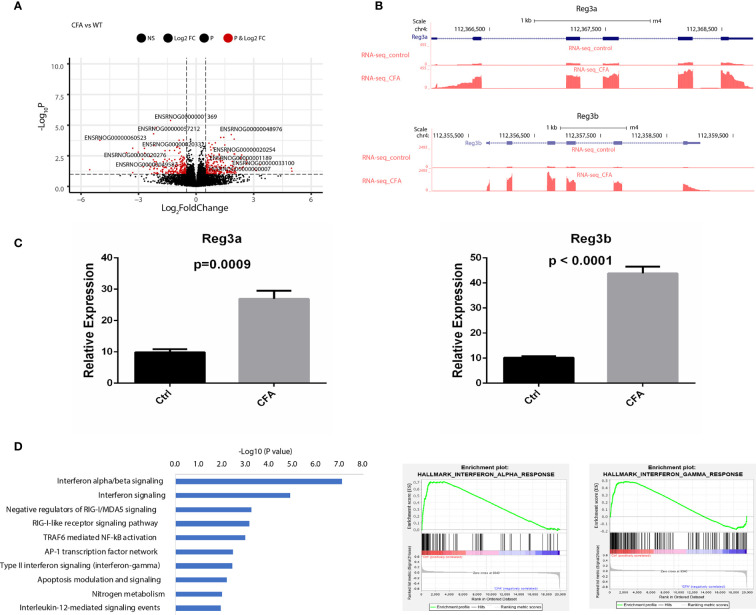
CFA-induced differential expression of inflammation-related genes. **(A)** Volcano plot showing the differentially expressed genes between CFA-treated and control rats. **(B)** Track plots showing the top 2 upregulated genes. **(C)** qRT-PCR experiments validated that both Reg3a and Reg3b gene expression were highly increased in the CFA treatment group compared with those in the control group. The p-values were calculated using Student’s t-test. **(D)** Pathway enrichment analysis performed with EnrichR (left) or GSEA (middle and right). Significantly enriched pathways were determined by a p-value of less than 0.05.

To investigate transcriptomic changes during CFA-induced inflammation, we performed total RNA-seq in DRG tissues obtained from 10 pairs of rats with or without CFA treatment and generated an RNA-seq dataset with high quality in terms of sequencing quality, duplication levels, adapter contamination, and mapping rates. Differential expression analysis revealed 213 and 205 genes were significantly upregulated and downregulated, respectively, in the CFA model compared with the control (**Figure 2A** and **Supplementary Figure 1**). Among all of the significant DEGs, Reg3b and Reg3a are the top 2 genes, with greater than 6-fold increases in expression level (**Figure 2B**). A quantitative real-time PCR (qRT-PCR) assay was also performed to validate the changes in mRNA expression (**Figure 2C**). The regenerating gene (Reg) family is known to play important roles in cell proliferation, migration, and the inflammatory response (29). To examine transcriptomic changes globally, we also performed pathway enrichment analysis based on all DEGs and observed the enrichment of many inflammation-related pathways, such as the interferon (IFN) alpha/beta signaling pathway, the retinoic acid-inducible gene I (RIG-1)-like receptor signaling pathway, the tumor necrosis factor (TNF) receptor associated factor 6 (TRAF6)-mediated nuclear factor kappa B (NF-κB) activation and AP-1 transcription factor network (**Figure 2D**). TRAF6 mediated NF-κB activity has previously been linked to CFA-induced inflammation (13, 30, 31). Altogether, these RNA-seq based results not only further validated the successful construction of a CFA-induced inflammation rat model but also revealed the specific transcriptomic alternations induced by CFA treatment in rats (**Supplementary Table 1**).

Alternative polyadenylation (APA) has been shown to regulate gene expression by regulating the interactions between miRNA and RNA molecules (32). To investigate the gene regulation mechanism underlying CFA-induced inflammation, we first profiled and compared the APA events between CFA-treated and control rats by DaPars. However, no significant APA events were detected (**Supplementary Table 2**), indicating that APA is irrelevant to the gene expression changes observed in CFA-treated rats.

### miRNA Profiling of the CFA-Induced Inflammation Rate Model

**Figure 3:**
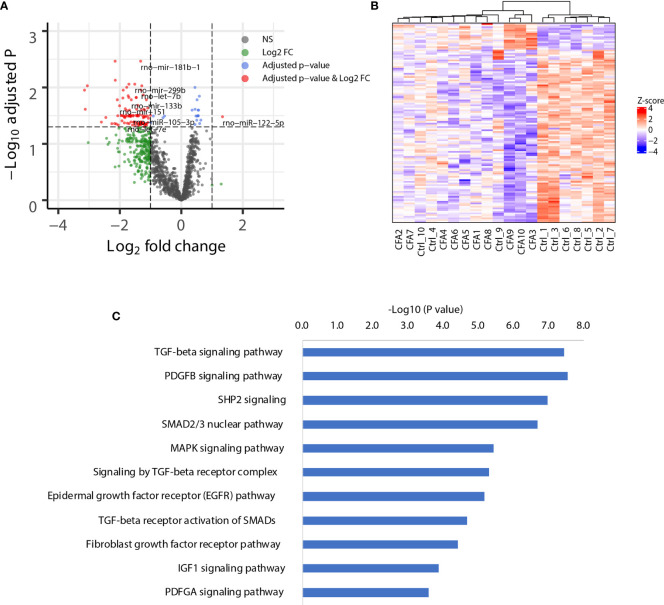
CFA-induced global downregulation of miRNAs targeting inflammation-related genes. **(A)** Volcano plot showing the global downregulation of miRNAs. **(B)** Heatmap plotted with significant miRNAs. Ward.D method and Euclidean distances were used for hierarchical clustering. **(C)** Pathway enrichment analysis of the genes targeted by the downregulated miRNAs, as assessed by EnrichR.

Although we did not observe APA changes in the CFA-treated rats, miRNAs might regulate gene expression directly through changes in the miRNA abundance. Several miRNAs have been reported to be associated with CFA-induced inflammation, including miR-134 in DRG tissue, and miR-124, miR-149, and miR-3584 in SDH tissue (16, 33). However, these studies did not include a comprehensive database, which should rely on a non-biased NGS platform. To provide a view of how the miRNA network is regulated following CFA treatment, we next performed small RNA-seq for the same 10 pairs of rat tissues obtained from rats treated either with or without CFA treatment. As expected, the differential expression analysis showed that 16 miRNAs were upregulated, including rno-miR-384-3p, which targets immune-related genes, such as like *Pik3cd*, *Nfatc3*, and *Cblb* (**Supplementary Table 3**). We also identified 104 significantly downregulated miRNAs, with the top being rno-miR-181b, which is predicted to target *Tnf* and *Irs2* (**Figure 3A**). The heatmap of miRNA expression profile also showed that control and CFA-treated rats were well-clustered (**Figure 3B**). To study the functions of the downregulated miRNAs in an unbiased manner, we performed pathway enrichment based on the miRNA target genes. Interestingly, many inflammation-related pathways were highly enriched in the CFA group, such as the transforming growth factor-beta (TGF-β) signaling pathway, the platelet-derived growth factor subunit B (PDGFB) signaling pathway, the cytoplasmic Src homology 2 (SH2) domain-containing phosphatase 2 (SHP2) signaling pathway, and the mitogen-activated protein kinase (MAPK) signaling pathway (**Figure 3C**). These results suggested that in response to CFA treatment, miRNAs are involved in the regulation of the inflammatory response.

### DNA Methylation Profiling of the CFA-Induced Inflammation Model in Rat

**Figure 4:**
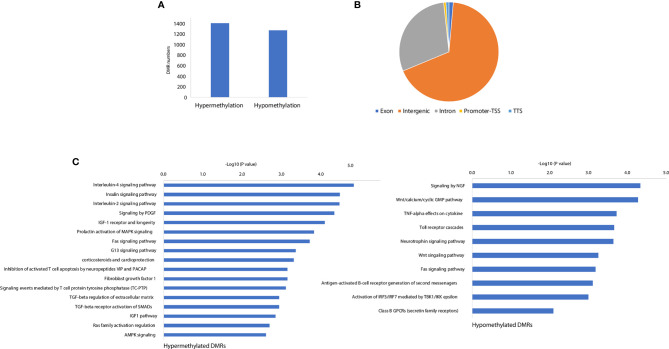
CFA-induced rewiring of the DNA methylation landscape. **(A)** The numbers of CFA-induced hypermethylated and hypomethylated DMRs. **(B)** Genomic annotations for the identified significant DMRs. **(C)** Pathway enrichment analysis for the DMR annotated genes by EnrichR.

Our previous work demonstrated that the promoter demethylation of the *CXCR4* gene was associated with CFA-induced inflammation (11). To further investigate the gene regulation mechanisms associated with the CFA-induced inflammatory response, we profiled the methylation landscape of CFA-treated rats by performing targeted bisulfite sequencing. Compared with the control rats, we identified a total of 2671 differentially methylated cytosines in the CFA-treated animals, suggesting that CFA treatment may have altered the DNA methylation landscape. We further performed DMR calling by using the ‘de-novo’ mode of metilene and identified 1,401 hypermethylated DMRs and 1,269 hypomethylated DMRs (**Figure 4A** and **Supplementary Table 4**). The majority of DMRs were enriched in the intergenic and intronic regions, with only 19 DMRs located in the promoter regions, suggesting that CFA-induced DNA methylation changes might not play significant roles in gene regulation through the direct repression of transcription at the gene promoter regions (**Figure 4B**). We then assigned DMRs to the nearest genes and performed pathway enrichment. For both hypermethylated and hypomethylated DMRs, we observed the enrichment of inflammation-related pathways, such as the IL-4 signaling pathway, the insulin signaling pathway, the TGF-β receptor activation of SMADs, and signaling through neural growth factor (NGF) and Toll-like receptor cascades (**Figure 4C**). Finally, we performed motif analysis with the DMRs and found the enrichment of the motifs for the transcription factors Tbp and Mtf1, both of which have previously been reported in association with the stress and inflammatory responses previously (34, 35). In summary, these results showed that DNA methylation was involved in the regulation of the CFA-induced inflammation response, likely by influencing the binding of inflammation-related transcription factors.

### AP-1 Works as a Potential Regulation Hub for the CFA-Induced Inflammatory Response

**Figure 5:**
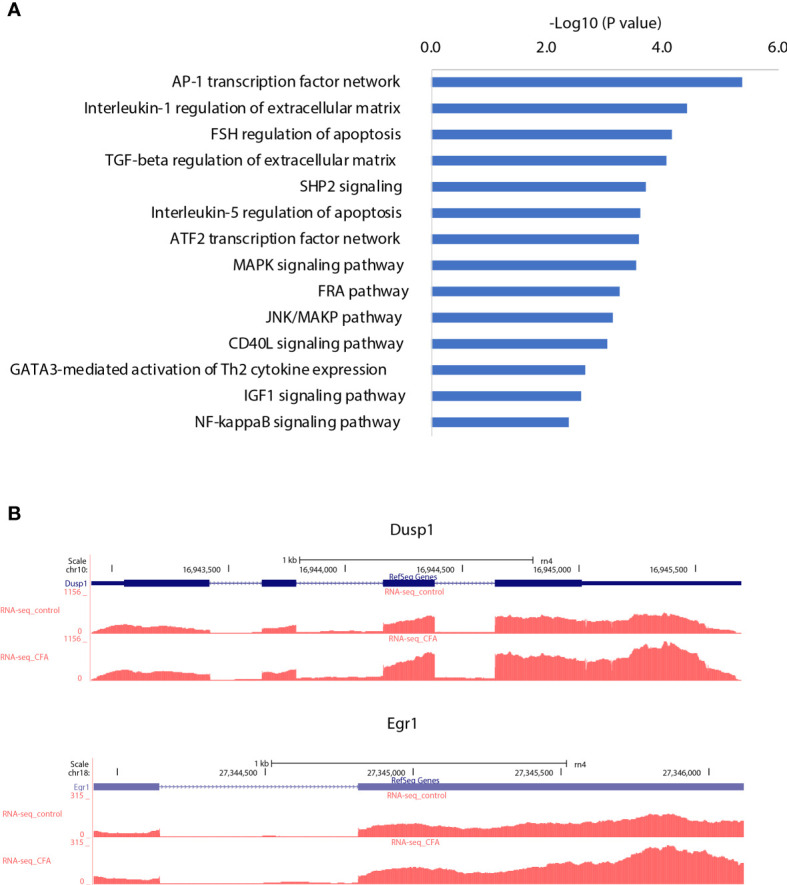
AP-1 transcription network may serve as a regulation hub integrating different types of gene regulation signals. **(A)** Pathway enrichment analysis for the target genes of the downregulated miRNAs, which were correspondingly upregulated. **(B)** Examples of AP-1 transcription network genes that were upregulated by CFA treatment.

**Figure 6:**
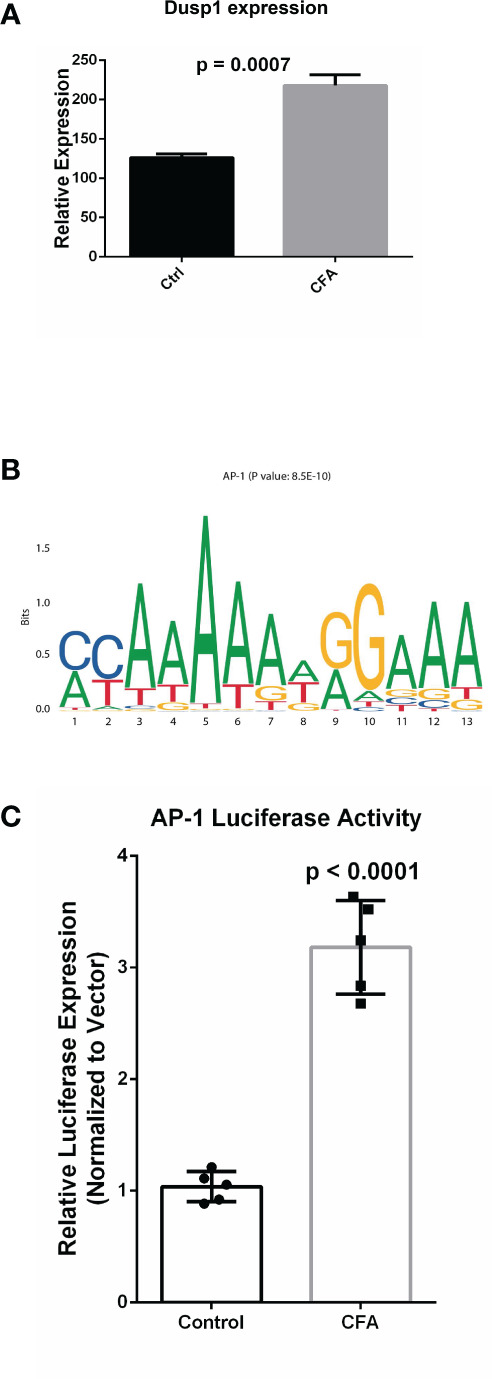
Experimental validation confirmed the upregulation AP-1 related gene in the response to CFA treatment. **(A)** qRT-PCR experiments demonstrated that the AP-1 related gene, *Dusp1*, was significantly increased in the CFA-treated group. All p-values are calculated from Student’s t-test. **(B)** Motif enrichment analysis for the DMRs by MEME-suite, showing the enrichment of the AP-1 binding motif. **(C)** Luciferase assays confirmed the upregulation of AP-1 activity in DRG tissues in CFA-treated rats compared with the control rats (p < 0.0001) by Student’s t-test.

After investigating CFA-induced changes in gene expression, miRNA, and DNA methylation, we examined the interactions among these components to illustrate how these 3 layers of molecular changes were coordinated. Because the majority of the differentially expressed miRNAs were downregulated, we focused on miRNA-targeted genes that displayed concomitant increases in their mRNA transcription levels (571 genes). These target genes were also enriched in many inflammation-related pathways, with the top pathway being the AP-1 transcription factor network (**Figure 5A** and **Supplementary Table 5**). The identified DEGs were also enriched in this pathway, further indicating that the miRNA-mediated repression of the AP-1 network was likely mitigated during the CFA-induced inflammation response (**Figures 2C** and **5B**, **Supplementary Table 6**). A validation experiment based on qRT-PCR also showed that *Dusp1*, an AP-1 related molecule, was significantly upregulated in the CFA group compared with the control group (**Figure 6A**). However, we failed to identify any differential expression for *Egr1* in the qRT-PCR results (**Supplementary Figure 2A**). Although the RNA-seq result exhibited a 1.5-fold upregulation in the Egr1 expression in the CFA group, most of the reads were enriched in the 3’ UTR, indicating a potential post-transcriptional regulation event. To determine whether *Egr1* was regulated by CFA treatment, we detected the protein expression level of Egr1 and found that Egr1 expression was slightly decreased in the CFA group (n =3, **Supplementary Figure 2b**). Compared with the upregulation in the mRNA level, this opposite trend observed for the protein expression may be a consequence of the increased expression of the 3’UTR. We also examined the interaction between DNA methylation and gene expression. Because the majority of DMRs were annotated to intergenic regions, we are not able to observe significant anti-correlations between differential methylation and differential expression. However, based on the motif analysis, we found that CFA-induced DMRs were surprisingly enriched for the AP-1 binding motif [**Figure 6B**, (**Supplementary Table 7**)]. Given that AP-1 is a well-known key regulator of the inflammatory response and has previously been linked to CFA-induced inflammation (36), we speculate that AP-1 may serve an important role as a hub factor, subjected to the regulation of both miRNA and DNA methylation following CFA treatment. Therefore, we performed luciferase assays to measure the AP-1 transcription activity in the DRG tissues of rats after CFA treatment. We found that AP-1 signaling was significantly increased in the CFA group compared with that in the control group (**Figure 6C**). This result directly confirmed our hypothesis that AP-1 serves an important role in response to the CFA treatment, contributing to the process of chronic inflammation.

## Discussion

**Figure 7:**
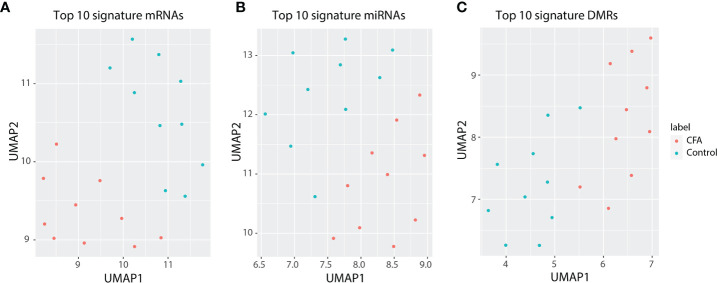
Top 10 differentially expressed genes can represent a signature for the precise separation between the CFA treatment and control groups. **(A)** UMAP for 10-signature genes of mRNAs. **(B)** UMAP for 10-signature miRNAs. **(C)** UMAP for 10-siganture DMRs.

The field of “omics” refers to the comprehensive assessment of a set of molecules. The integrated analysis of multiple NGS-based datasets, which highly rely on high-throughput sequencing, has already revolutionized the medical research (37). Chronic pain is a critical health issue globally, affecting millions of people. However, directly obtaining tissues from patients can be challenging. Thus, we believe that examining the molecular networks of chronic pain in animal models using a comprehensive approach represents a suitable alternative to the study of human tissues. We provided an integrated analysis based on the molecular profile obtained for the response to CFA injections in rat DRG tissues, at the epigenetic, transcriptional, and post-transcriptional regulation level. Overall, we identified 418 differentially expressed mRNAs, 120 differentially expressed miRNAs and identified more than 2,500 DMRs in the CFA-treated group relative to the control group. Through GSEA, we validated some of the previously reported CFA-response-related signaling pathways that are also highly associated with the CFA-induced inflammatory response, such as the NF-κB (13) and IFN signaling pathways (38). Moreover, we also identified many new genes and pathways that might be potentially involved in this pain response model, including Reg family genes (Reg3a, Reg3b) and the AP-1 transcription-related pathways. After we adjusted the cell heterogeneity of the methylation sequence data by using CHALM (39), a recently developed software program for analyzing the methylome, we identified 6,832 significant DMRs, suggesting that CFA’s impact on the rat methylome is underestimated by the traditional analysis methods. In addition to inflammation-related pathways or terms, the CHALM program identified DMRs enriched in pathways associated with heart contractions and heart rate, indicating that methylome rewiring may be involved in chronic pain-associated heart malfunctions or diseases (**Supplementary Table 8**). Finally, based on our multi-omics profiling of the CFA-induced chronic pain rat model, we selected the top 10 DEGs, miRNAs, and DMRs to represent the multi-omics signature of our chronic pain model. The CFA treatment and control groups could be clearly separated by these signatures (**Figures 7A–C** and **Supplementary Tables 9–11**). These findings were consistent with the idea that miRNA and DNA methylation regulate mRNA transcription.

Pancreatitis-associated proteins, which are derived from Reg families, have previously been previously linked to the modulation of spinal sensory pathways in pathological pain states (40). Our results demonstrated that Reg3b and Reg3a were significantly upregulated, by 12-fold and 6-fold, respectively, in the CFA-treated group compared with the control group, indicating that Reg family genes may play a crucial role in the regulation of the inflammatory pain response in DRG tissues. The AP-1 transcription factor contains a dimerized bZIP (basic region leucine zipper) domain, *via* the Fos and Jun subunits. The AP-1 regulation network was previously linked to the chronic pain response (36) but was not previously recognized as a central pathway in the pain response. In our dataset, the AP-1 network was considered to serve as the central regulation hub. The CFA-induced miRNA and mRNA interactions were highly enriched for AP-1 networks, and the CFA-induced DMR motifs were enriched in AP-1 transcription factor binding activities. Therefore, we hypothesized that the inhibition of certain AP-1 network genes, such as *Egr1*, which is recognized as both a pleiotropic inflammatory transactivator (41) and a chronic pain contributor (42), could potentially serve as a mechanism for the alleviation of chronic pain. Interestingly, most of the differentially expressed miRNAs (104/120) were downregulated after CFA treatment. The global downregulation of miRNAs is itself an interesting phenomenon, which was missed in the previous array-based study (16). Notably, among these downregulated miRNAs, many target the AP-1 network genes. Thus, we speculate that supplementation with these miRNAs could inhibit the overactivation of the AP-1 network and alleviate pain. Overall, we believe that the AP-1 network plays a central role in the regulation of inflammatory pain responses through a methylation-transcription-posttranscriptional regulation axis.

In conclusion, this study provided a comprehensive transcriptomic profile of the CFA-induced inflammatory pain rat model *via* an approach that targeted DNA methylation, gene expression, and post-transcriptional regulation. Our study has certain limitations. First, although we included 10 pairs of rats for CFA treatment, we only examined a single time point following CFA injection, which was 24 hours. Future studies should focus on 48 hours, 72 hours, or even 7 days post-treatment, as CFA could also induce chronic inflammation. In addition, although we demonstrated that AP-1 is likely to serve as a regulatory hub for the CFA-induced inflammation response, we did not perform sufficient biochemical assays to further investigate alterations in the AP-1 signal regulation. Future studies could focus on the effects of individual molecules or genes within the AP-1 network in the regulation of chronic pain. Finally, another limitation of this study is that we only utilized male mice in this study. This is because a previous study showed that there exists a different pain hypersensitivity in female and male mice depends on different types of immune cells (43). Sorge et al. originally found that inflammatory and neuropathic pain in male, but not the female mice depended on the Toll-like receptor 4 (TLR4) (44). In order to reduce the potential variation of the pain measurement, we decided to utilize only male mice in this study. Future studies should also focus on the chronic pain in the female mice undoubtedly.

## Conclusion

Our study provides the first comprehensive molecular profile of the CFA-induced inflammatory pain model using an integrated omics approach. Our results revealed that the AP-1 signaling pathway may play a central role in the regulation of pain-related signaling.

## Data Availability Statement

The original contributions presented in the study are publicly available. This data can be found here: https://www.ncbi.nlm.nih.gov/geo/query/acc.cgi?acc=GSE174631.

## Ethics Statement

The animal study was reviewed and approved by Suzhou Municipal Hospital.

## Author Contributions

LW designed the research and analyzed the data. FL, MW, HS, XW, HQ, WZ, CS, and CW performed research and analyzed data. XZ and LW wrote the paper. All authors contributed to the article and approved the submitted version.

## Funding

This study was supported by the National Natural Science Foundation of China [NSFC 81701105 and 31872773] and the Postdoctoral Research Funding Program of Jiangsu Province [2018K257C].

## Conflict of Interest

The authors declare that the research was conducted in the absence of any commercial or financial relationships that could be construed as a potential conflict of interest.

## Abbreviations

CFA, Complete Freund’s Adjuvant; miRNA, microRNA; DRG, dorsal root ganglion; SDH, spinal cord dorsal horn; SD rat, Sprague Dawley rat; FDR, false discovery rate; PWT, paw withdrawal threshold; PWL, paw withdrawal latency; DEGs, differentially expressed genes; APA, alternative polyadenylation; DMR, differentially methylated regions.
