# Supplementary figures and images for: Integrated Analysis of Omics Data Reveal AP-1 as a Potential Regulation Hub in the Inflammation-Induced Hyperalgesia Rat Model
Source: Front Immunol. 2021 May 28;12:672498. doi: 10.3389/fimmu.2021.672498 (PMC8194263; doi:10.3389/fimmu.2021.672498)

Supplementary Figure 1

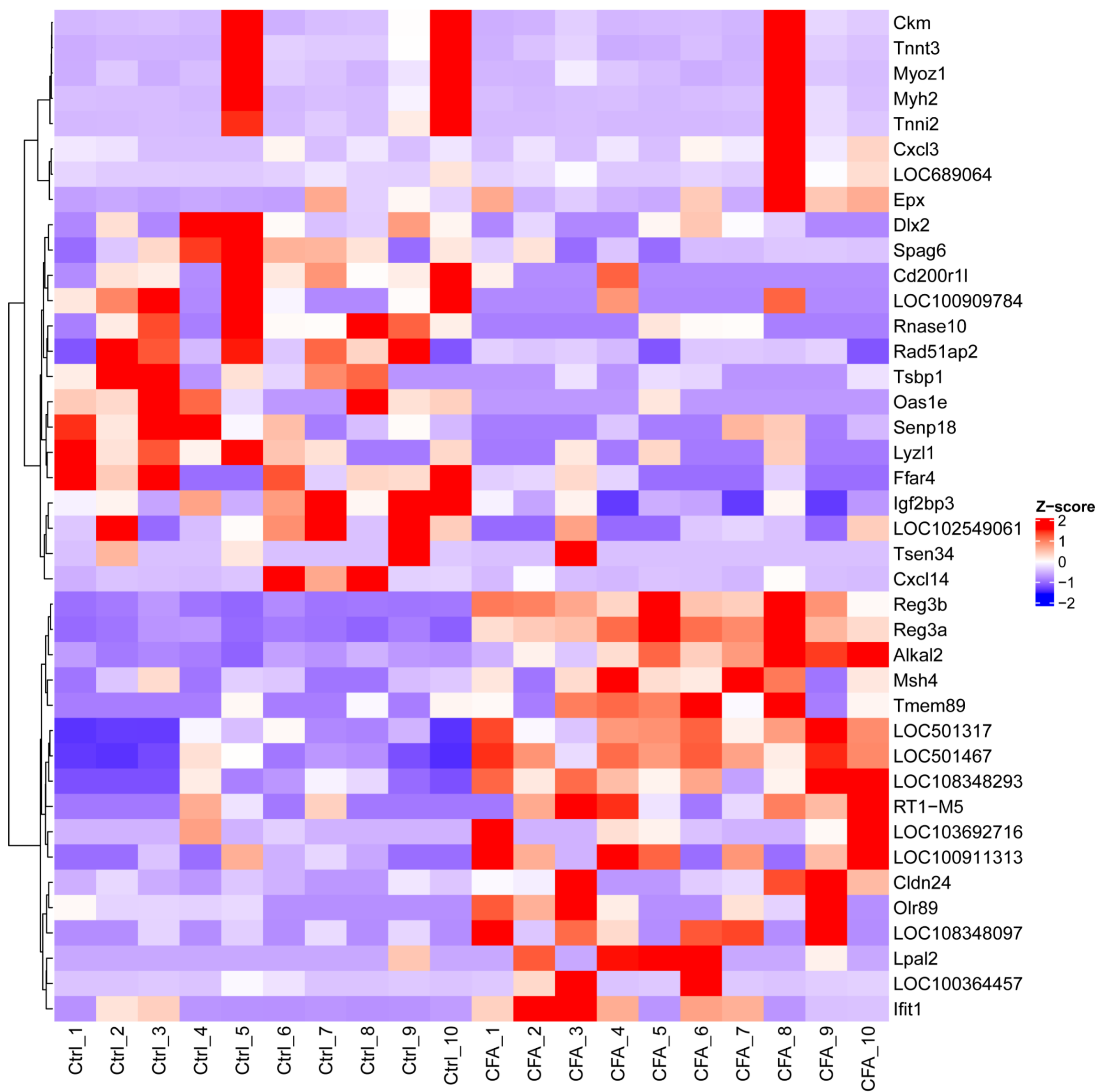

# Supplementary Figure 2

## a Egr1 expression

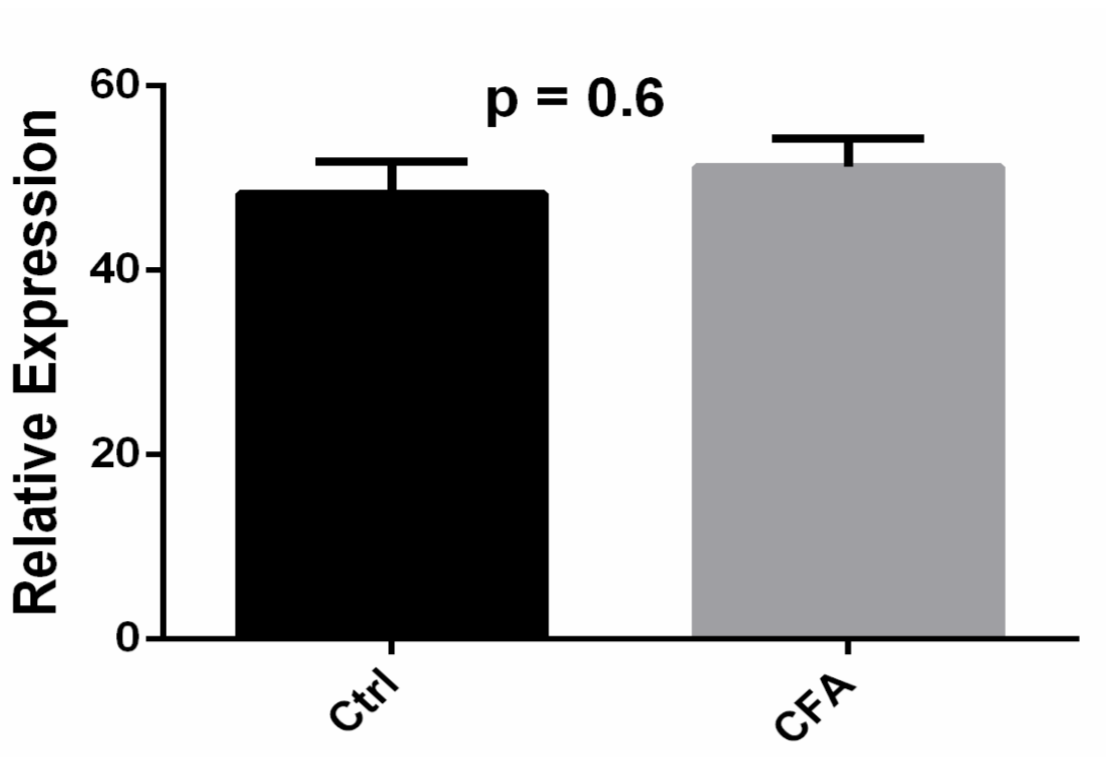

## b

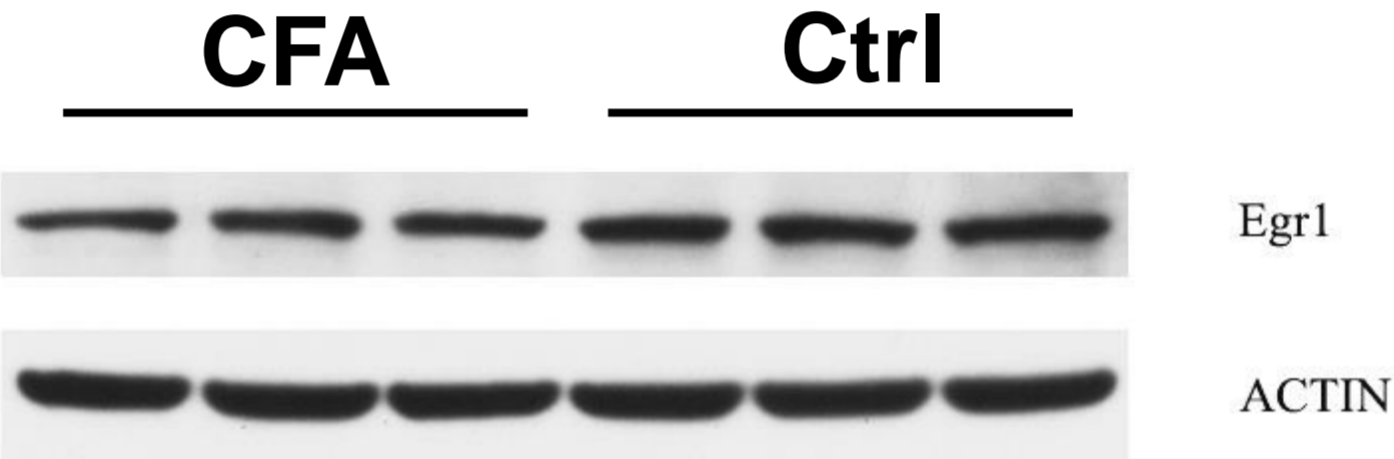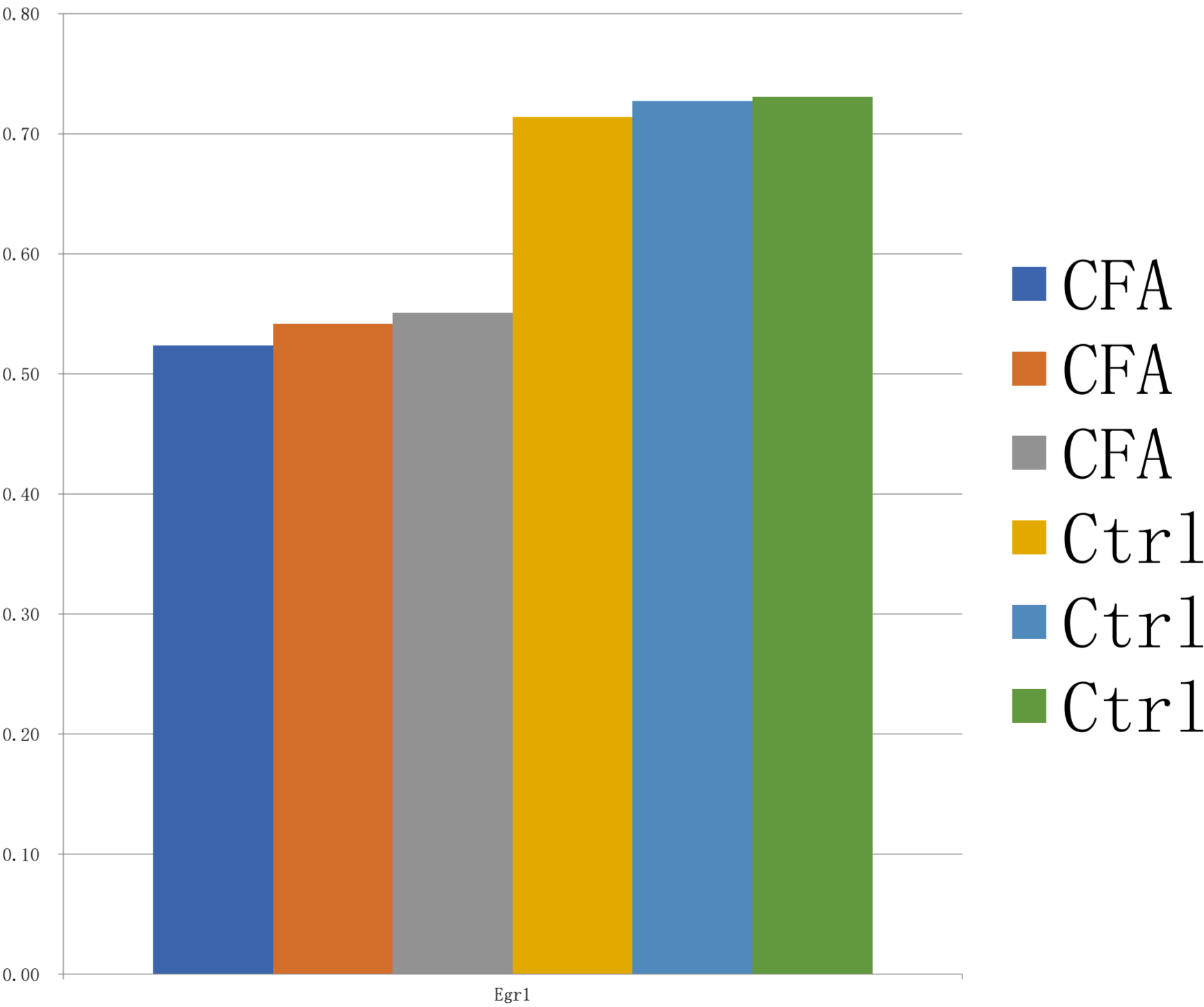

Supplementary Figure 3

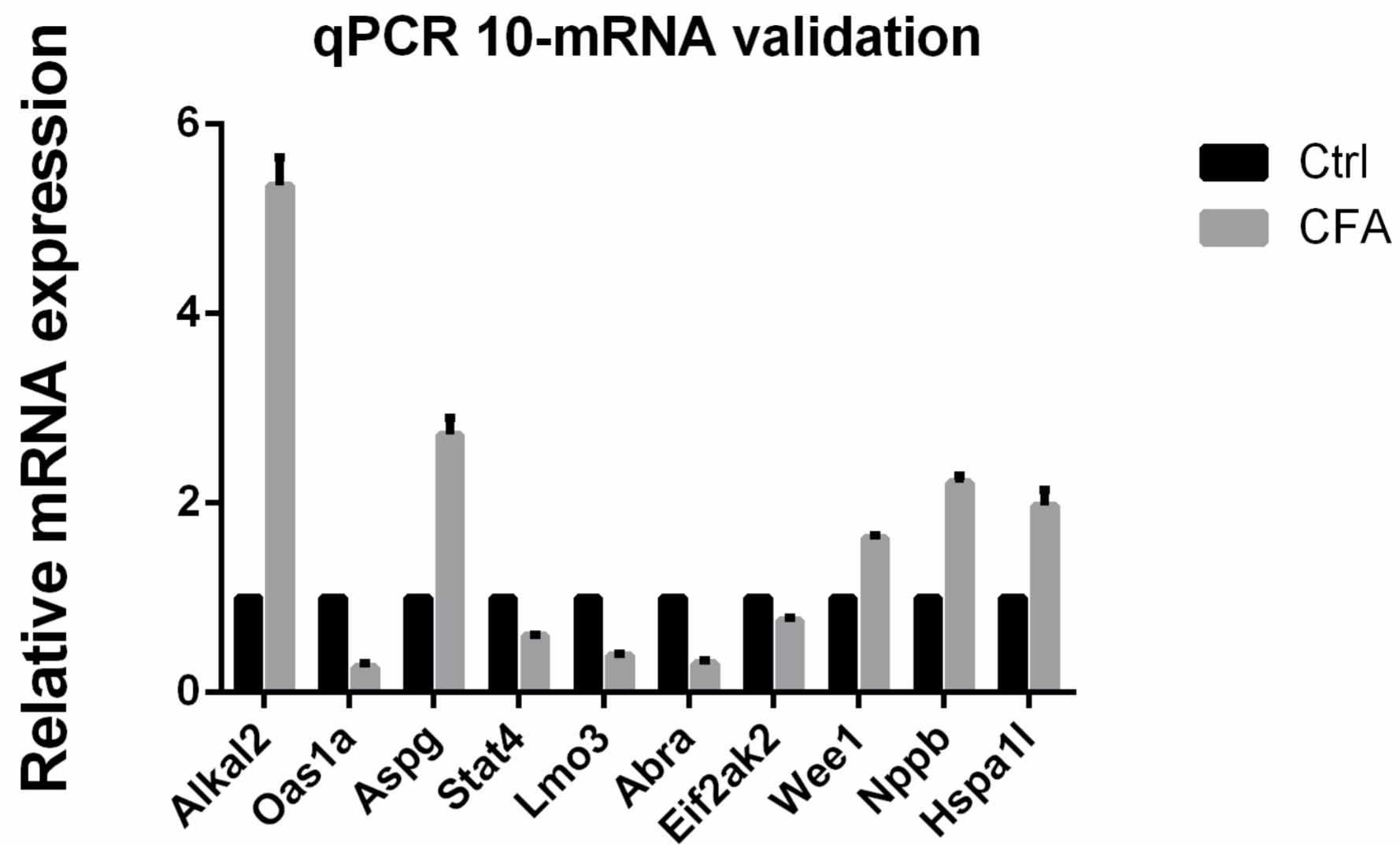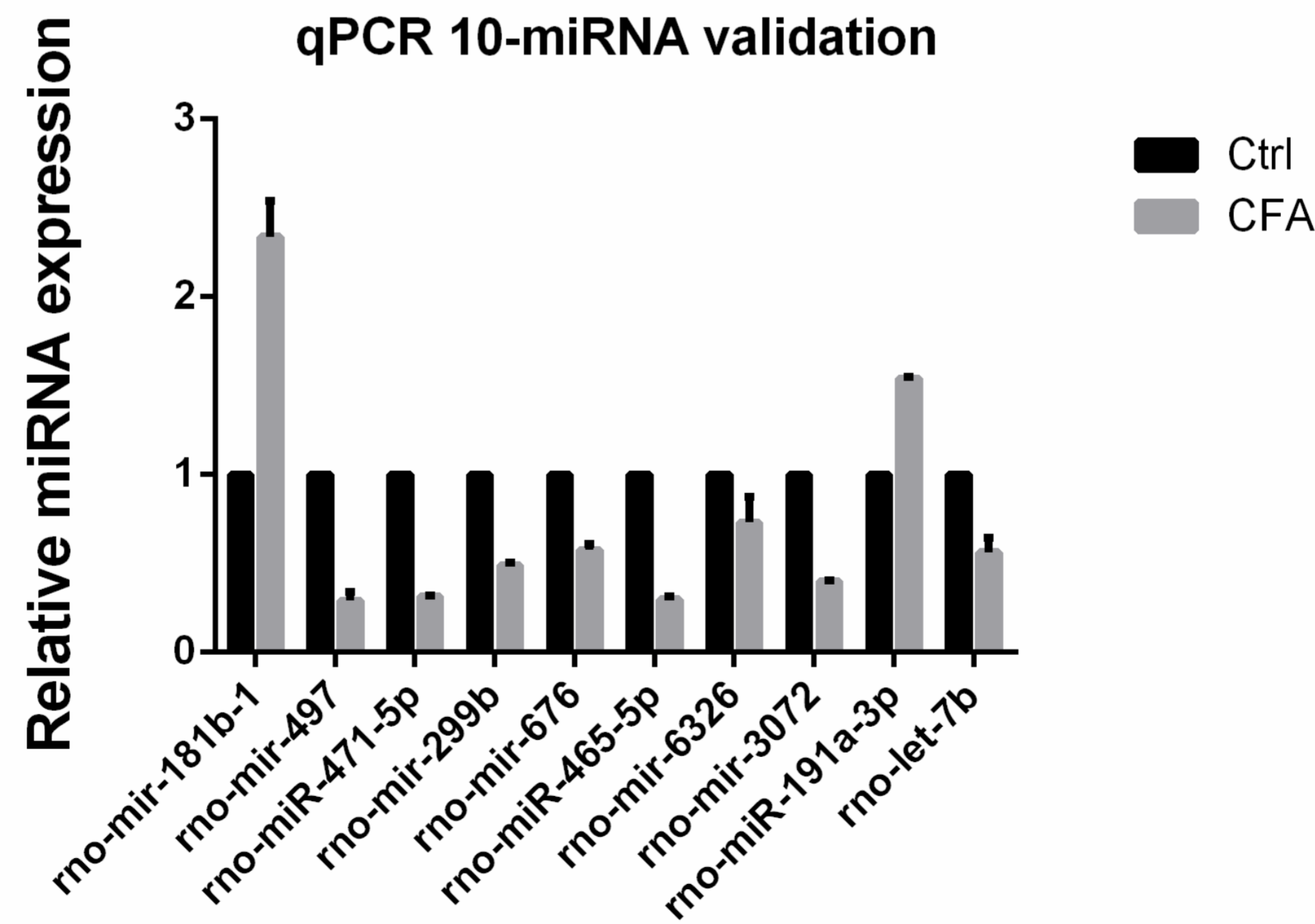

Supplement: Supplementary file 1 [file Image_1.pdf]
